# Regional Grey Matter Structure Differences between Transsexuals and Healthy Controls—A Voxel Based Morphometry Study

**DOI:** 10.1371/journal.pone.0083947

**Published:** 2013-12-31

**Authors:** Lajos Simon, Lajos R. Kozák, Viktória Simon, Pál Czobor, Zsolt Unoka, Ádám Szabó, Gábor Csukly

**Affiliations:** 1 Semmelweis University, Department of Psychiatry and Psychotherapy, Budapest, Hungary; 2 Semmelweis University, Magnetic Resonance Imaging Research Center, Budapest, Hungary; 3 Nathan Kline Institute for Psychiatric Research, Orangeburg, New York, United States; Beijing Normal University, China

## Abstract

Gender identity disorder (GID) refers to transsexual individuals who feel that their assigned biological gender is incongruent with their gender identity and this cannot be explained by any physical intersex condition. There is growing scientific interest in the last decades in studying the neuroanatomy and brain functions of transsexual individuals to better understand both the neuroanatomical features of transsexualism and the background of gender identity. So far, results are inconclusive but in general, transsexualism has been associated with a distinct neuroanatomical pattern. Studies mainly focused on male to female (MTF) transsexuals and there is scarcity of data acquired on female to male (FTM) transsexuals. Thus, our aim was to analyze structural MRI data with voxel based morphometry (VBM) obtained from both FTM and MTF transsexuals (n = 17) and compare them to the data of 18 age matched healthy control subjects (both males and females). We found differences in the regional grey matter (GM) structure of transsexual compared with control subjects, independent from their biological gender, in the cerebellum, the left angular gyrus and in the left inferior parietal lobule. Additionally, our findings showed that in several brain areas, regarding their GM volume, transsexual subjects did not differ significantly from controls sharing their gender identity but were different from those sharing their biological gender (areas in the left and right precentral gyri, the left postcentral gyrus, the left posterior cingulate, precuneus and calcarinus, the right cuneus, the right fusiform, lingual, middle and inferior occipital, and inferior temporal gyri). These results support the notion that structural brain differences exist between transsexual and healthy control subjects and that majority of these structural differences are dependent on the biological gender.

## Introduction

Transsexualism is a heterogenous condition both in its manifestation and etiology. There are numerous chromosomal abnormalities or well-defined biological causes that can lie behind the incongruence between an individual's biological gender (i.e. that based on the sex chromosomes and/or the manifestation of the sexual organs) and gender identity (i.e., someone's sense and perception of being male or female). However, there is a group of individuals who do not show such known genetic or somatic abnormality and yet experience strong incongruency between their assigned biological gender and their gender identity. In the Diagnostic and Statistic Manual of Mental Disorders 4th edition - Text Revision [1] this is referred as Gender Identity Disorder (GID). GID is characterized by a long-standing and strong feeling of being a member of another gender, long-standing distress about the assigned gender and feeling of incongruity with the assigned gender-roles causing significant clinical discomfort and impairment in both the individual's social and professional life and in other life areas. Based on DSM-IV-TR, GID cannot be diagnosed if transgender experiences are associated with any physical intersex conditions.

The background of transsexualism has been the topic of debate for decades. Recently, mainly early developmental disturbances have been suggested by the pertinent literature [2]. According to a recent review about the sexual differentiation of the human brain, transsexualism might be the result of the fact that the development of the sexual organs in the fetal life occurs well before the sexual differentiation of the brain. Thus, if something disturbs the sexual differentiation of the brain, the fetus already has sexual organs according to his/her assigned sex, while his/her brain might develop differently [2]. These authors suggest that the disturbance of the testosterone surge that masculinize the fetal brain might be at the background of GID in certain cases. Furthermore, they emphasize that there is no compelling evidence that postnatal environmental factors play a crucial role in sexual orientation and gender identity [2]. The theories about the neurobiological background of GID were partly based on earlier neuroanatomical findings of the same group, Swaab and collegues. This group described differences in the brain structure of Male to Female (MTF) and Female to Male (FTM) transgender subjects and controls (post mortem) in regions of the brain that showed sex differences regarding their size [3]. Specifically, they found that the size and the number of neurons in the bed nucleus of striata terminalis (BSTc) and the third interstitial nucleus of the anterior hypothalamus (INAH-3) of MTF transgender patients were typical for the size and neuron numbers found generally in females [3]–[5].

In the last two decades, however, structural imaging studies reported controversial results about structural brain differences in transgender and control subjects. Four structural MRI studies focused on white matter differences in transgender patients. Emory et al. [6] found no difference in the whole corpus callosum or the splenium region between transsexuals and controls. More recently, Yokota et al. [7] concluded that the pattern of corpus callosum shape in transsexuals was closer to that in subjects with the same gender identity than to that in subjects with the same biological sex. Rametti et al. conducted two separate studies in untreated FTM and MTF patients, applying diffusion tensor imaging (DTI) [8], [9]. They found that in case of FTM transsexuals certain fasciculi involved in higher cognitive functions the white matter microstructure pattern was closer to the pattern of subjects who shared their gender identity (males) than to those who shared their biological gender (females) [8]. In case of MTF transsexuals, DTI findings showed that the white matter microstructure pattern was intermediate between male and female controls. Based on the direction of the differences the authors suggested that certain fasciculi did not complete the masculinization process during brain development [9]. Rametti et al. (2012) [10] further investigated FTM transsexuals in order to examine the effect of testosterone treatment on brain structure. They found that testosterone treatment changed white matter microstructure in FTMs. Specifically, the fractional anisotropy (FA) values in the superior longitudinal fascicule and the right corticospinal tract increased after 7 months of testosterone treatment, compared with the FA values before treatment. Furthermore, this effect was predicted by the pretreatment, free testosterone levels [10].

Four structural MRI studies investigated the structure of grey matter (GM) and yielded in mixed results. One study, using voxel based morphometry (VBM) concluded that the right putamen was “feminized” in MTF transsexuals, another study applying VBM and MR volumetry concluded that their data did not support the hypotheses according to which MTF transsexuals show feminized brain structure in certain areas, however reported on particular features of the brain structure of nonhomosexual MTF transsexuals including areas that are involved in body perception [11], [12]. The third study, measuring GM thickness reported thicker GM in MTF transsexuals compared with control males in several brain areas both in the left and the right hemisphere, along both the medial and lateral surface of the cortex [13]. This study concluded that their results support the notion that gender identity and brain anatomy are associated and that a “shift” exists between MTF transsexuals and gender congruent males with regard to brain structure [13]. The most recent study in this field investigated cortical thickness and volumetric differences of certain subcortical regions in both MTF and FTM transsexuals by MR volumetry. This study found that FTMs showed subcortical grey matter masculinization (right putamen), while MTFs showed feminization with regard to cortical thickness, as well as right hemisphere localized differences compared with male controls [14].

Except for the most recent study of Zubiaurre-Elorza et al (2012) [14], all of the aforementioned three studies obtained their data only on MTF transsexuals. Thus, there is scarcity of imaging data on FTM transsexuals. Our objective was to compare the regional cortical structure of both untreated MTF and FTM transgender subjects with that of male and female control subjects, applying VBM.

Our hypothesis was that the regional structural parameters of the brain of transsexual subjects will be different from that of control subjects with the same biological gender.

## Methods

This investigation was carried out in accordance with the latest version of the Declaration of Helsinki. The study was approved by the local ethics committee (Semmelweis University Regional and Institutional Committee of Scientific and Research Ethics) and all included subjects provided written informed consent.

### Subjects

All consecutively arriving patients of the Transgender Special Outpatient Service of the Psychiatry and Psychotherapy Department of the Semmelweis University (Budapest, Hungary) diagnosed with GID, based on the DSM-IV TR diagnostic criteria [1] were approached to enter a neuroimaging study, between 2007 September and 2009 March. Both MTF and FTM patients were eligible for the study, but only those with homosexual orientation. The rationale for this choice was based on the Blanchard typology [15] which considers two fundamentally different types of transsexualism: homosexual and nonhomosexual. Homosexual transsexual individuals are sexually attracted to the same biological gender, while nonhomosexual transsexual individuals are attracted to either the opposite gender or show no sexual orientation/attraction at all. According to Blanchard, homosexual transsexuals are usually younger at initial presentation of gender identity disorder and show more pronounced and frequent childhood femininity, as well as different anthropometric data [16], [17]. One might argue that mixing individuals from both transsexual groups in one study targeting the neurobiological background of transsexualism might bias the results by introducing heterogeneity in the sample. Thus, in our study, only homosexual transsexual individuals were included preventing our findings from the aforementioned bias. Evaluation of the subjects' sexual orientation was based on self-report.

Further exclusion criteria were: previous cross-hormonal treatment, any known chromosomal or hormonal disorder in the background of transgender identity, any neurological disorder in the anamnesis.

Healthy volunteers were recruited to serve as controls from among medical students, colleagues and friends of the research team who were free from any symptoms of GID or any psychiatric disorders. The presence of symptoms of GID was evaluated based on a free clinical interview asking simple questions targeting the symptoms of GID listed in DSM-IV-TR, while the presence of psychiatric symptoms was assessed by SCL-90 [18]. Control subjects were selected to represent a population matched in age and gender identity to the patient group.

Only data from the structural imaging findings are presented in this paper, results of the functional imaging findings will be reported in upcoming publications.

### Diagnosis of GID

All GID subjects underwent a detailed diagnostic interview with an expert psychiatrist in the field and also filled out a test battery assessing transgender identity disorder symptoms and associated behaviors and psychiatric comorbidity in order to confirm the diagnosis and exclude the presence of other mental disorder behind the symptoms of GID. Sexual orientation of the patients was assessed by self-report. During the clinical interview basic demographic data, family history, psychiatric history and psychiatric status were also collected.

### 2.3 MRI image acquisition

High resolution anatomical images of all participants were collected at the MR Research Center (Semmelweis University, Budapest, Hungary) on a 3 Tesla Philips Achieva whole body clinical MRI scanner (Philips, Best, The Netherlands) equipped with an 8-channel SENSE head-coil. Whole brain anatomical images were obtained using a T1 weighted three dimensional spoiled gradient echo (T1W 3D TFE) sequence as provided by the vendor, but fine-tuned to provide the best possible separation between white matter and GM; 180 contiguous slices were acquired from each subject with the following imaging parameters: TR = 9.7 ms; TE = 4.6 ms; flip angle  = 8°; FOV of 240 mm×240 mm; voxel size of 1.0×1.0×1.0 mm.

### Data processing

We performed voxel based morphometry analysis [19] on the imaging data to investigate differences in local GM volume between our subject groups. Data preprocessing and analysis were performed within the SPM8 software framework (http://www.fil.ion.ucl.ac.uk/spm/) using the VBM8 toolbox (http://dbm.neuro.uni-jena.de/vbm/). We applied the default processing parameters of the VBM8 toolbox using the default preprocessing pipeline consisting of the following steps: (a) segmentation of the different tissue classes, (b) linear (i.e., affine) and nonlinear (i.e., Dartel) registration [20] of the subjects' brains to the Montreal Neurological Institute (MNI) atlas space, [21] with 1.5×1.5×1.5 mm resolution using the ICBM152 and DARTEL templates supplied with the SPM8 toolbox and (c) modulation of the GM tissue segments by the nonlinear normalization parameters to account for individual brain size differences. The segmentation procedure was refined by accounting for partial volume effects [22], applying adaptive maximum a posteriori estimations, and denoising using a hidden Markov random field model [23], and also by using a spatially adaptive non-local means filters [24]. Next, the whole data set was evaluated for outliers by checking sample homogeneity using covariances between image pairs; none of the images were excluded from further analysis based on this check. Finally, the realigned and normalized GM segments were smoothed with an 8 mm FWHM Gaussian kernel.

### Statistical analysis

Voxel-wise image intensities (henceforth GM intensities) of the smoothed warped modulated GM compartments representing regional GM volume were compared using a 2x2 ANCOVA model specified in SPM8, with Biological gender, and the presence of GID as main effects and age as a covariate of no interest. Upon whole brain model estimation, F contrasts were calculated to assess the sites of main effects and interactions (GID, Biological gender and GID × Biological gender). Statistical maps were considered significant at the level of p<0.001 uncorrected, due to the limited statistical power we present unthresholded F-statistical maps along with thresholded maps, to show that the significant clusters are indeed markedly different from the background [25]. To limit the possibility that local image artifacts or noise affect the outcome of statistical comparisons a cluster size threshold of 30 voxels was set; this way only clusters with a minimum volume of 101.25 cubic millimeters were considered relevant. Anatomical labeling of significant clusters was performed using the xjView toolbox (http://www.alivelearn.net/xjview).

Post hoc analyses were performed on significant clusters for the Biological gender, GID, and GID × Biological gender interaction contrasts. To this end, GM intensity values corresponding to the statistically significant clusters were extracted from the individual brains, and averaged within subject using the MarsBaR SPM toolbox (http://marsbar.sourceforge.net/) [26] and processed further in Matlab as follows: in case of the Biological gender and GID contrasts post hoc F and T statistics were calculated; for these comparisons differences were considered significant at a level of p<0.05. In case of clusters of the GID × Biological gender contrast a 2×2 ANOVA was performed, followed by all possible pairwise comparisons of the control females, control males, FTM transsexuals and MTF transsexuals, using unpaired two-sample Student's tests between the groups. The significance levels for these post hoc Student's tests were corrected for multiple comparisons using the Bonferroni method, i.e., only results with p<0.05/6 = 0.008 (as six comparisons were performed for a given cluster), were considered as significant.

## Results

### Basic demographic data

Seventeen patients with the diagnosis of GID (10 MTF patients and 7 FTM patients), based on the DSM-IV TR [1] diagnostic criteria, before cross-hormonal treatment, and 18 age matched control subjects (11 females and 7 males) were included in the study. Mean age of subjects with GID was: 28.5 years (SD:7.69) for males and 24.8 years (SD:6.45) for females, while mean age of controls was 27.1 years (SD: 5.54) for males and 23.9 years (SD:3.42) for females.

### 3.2 Results of whole brain VBM

**Figure 1:**
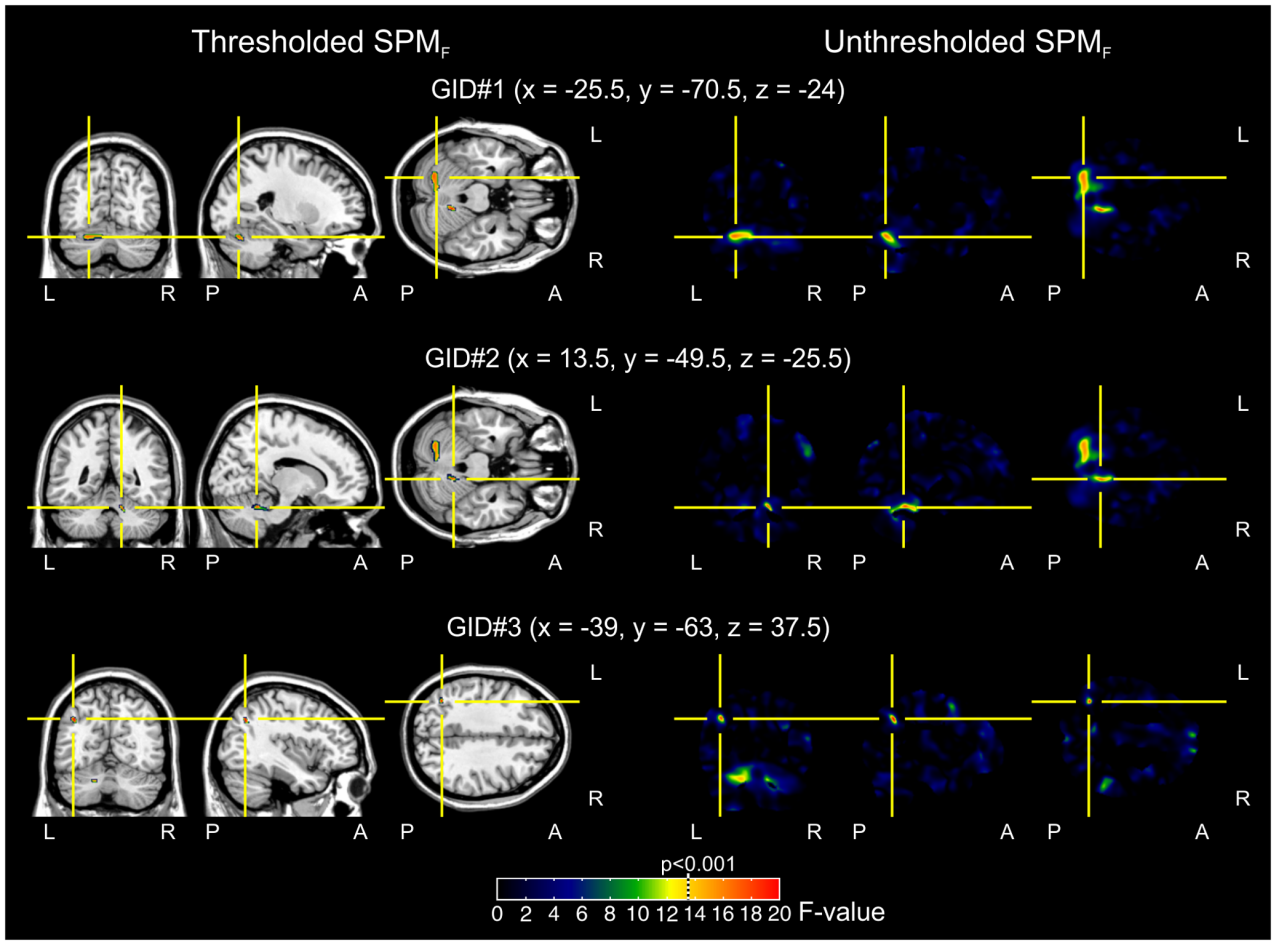
Main effect of GID. The figure represents clusters with significant GM volume difference, depending on GID status. The results are based on a group-wise 2×2 ANCOVA model, estimated upon the whole brain, where GID status and Biological gender were the main factors and age served as a covariate of no interest. Differences were considered significant at p<0.001, uncorrected with a cluster size threshold of 101.25 cubic millimeters (left panels). In the right panels the unthresholded SPM_F_ maps are shown. Color coding of clusters in the left panels and maps in the right panels is based on F-values, and is similar across clusters; the p<0.001 threshold is marked with a dotted line on the color bar. GID: Gender Identity Disorder; GM: grey matter

**Figure 2:**
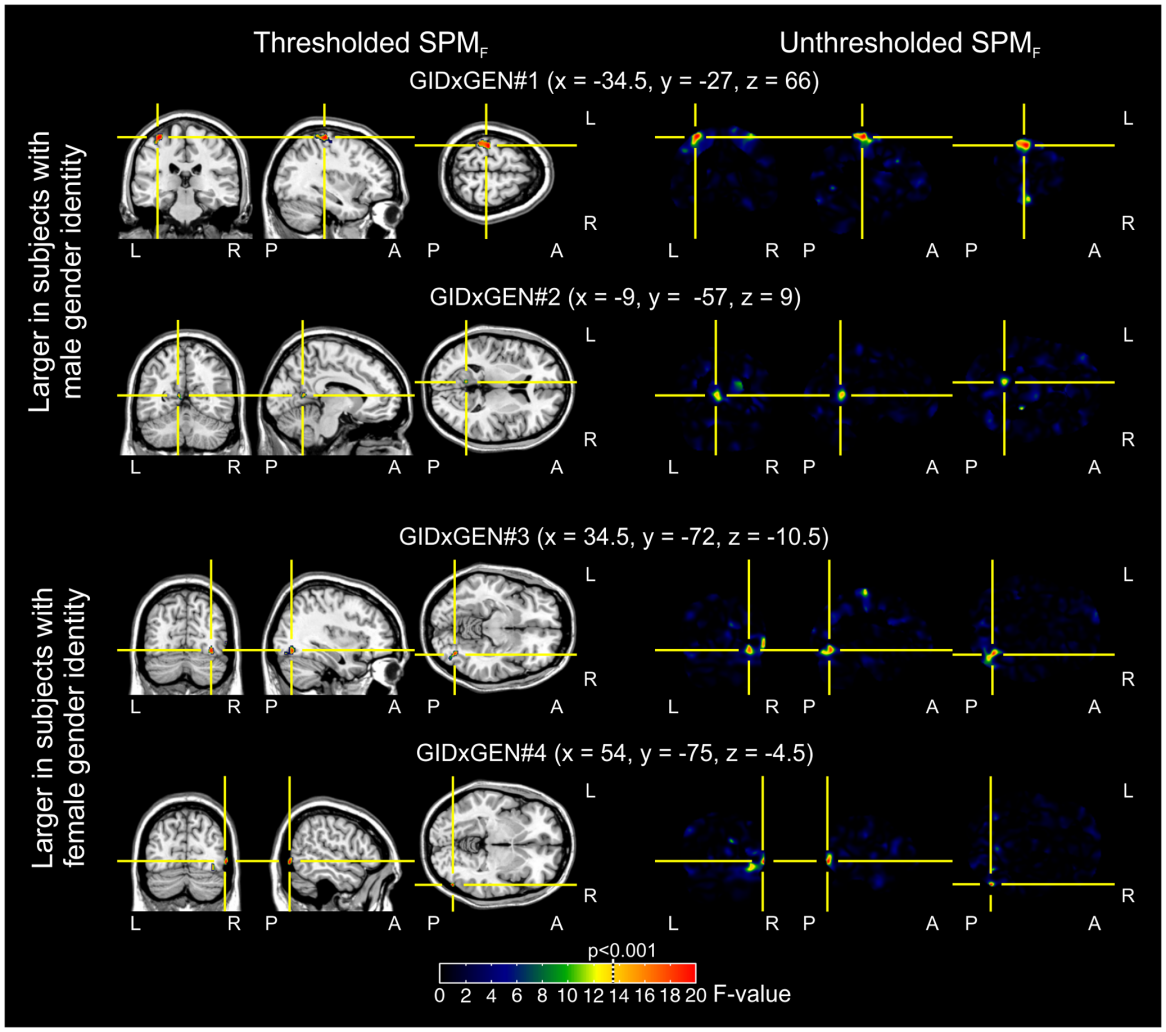
The sites of GID × Biological gender interaction. Clusters with significant GID × Biological gender interaction are shown, based on a 2×2 ANCOVA model where age served as a covariate of no interest. Interactions were considered significant upon whole brain model estimation at p<0.001 uncorrected with a cluster size threshold of 101.25 cubic millimeters (left panels). In the upper rows, those clusters are visible in which GM volume was larger in subjects with male gender identity, while the lower rows depict clusters in which GM volume was larger in subjects with female gender identity. In the right panels, the unthresholded SPM_F_ maps are shown. Color coding of clusters in the left panels and maps in the right panels is based on F-values, and is similar across clusters; the p<0.001 threshold is marked with a dotted line on the color bar. GID: Gender Identity Disorder; GEN: Biological gender; GM: Grey matter

The whole brain analysis showed 3 significant clusters for the GID effect, i.e. where the regional GM volume was found to be significantly different between patients and controls (Table 1 and Figure 1). The Biological gender main effect, accounting for gender differences but not for GID status was significant in 6 clusters (Table 2). Significant GID × Biological gender interactions were found in 4 clusters (Table 3, and Figure 2).

**Table 1:** Significant effect of GID on regional cortical structure^a^.

Cluster name	Cluster peak [x,y,z]^b^	Localization^c^	Number of voxels	Post hoc statistics F(1,30)	Post hoc statistics t(33)	Direction of main effect
GID#1	−25.5, −70.5, −24	Left Cerebellum Anterior and Posterior Lobe, Declive, Dentate	229	17.25*	4.376*	Larger in controls (#1–3)
GID#2	13.5, −49.5, −25.5	Right Cerebellum Anterior Lobe, Culmen, Dentate	76	19.75*	4.567*	
GID#3	−39, −63, 37.5	Left Angular Gyrus, Left Inferior Parietal Lobule	32	22.10*	4.877*	

^a^ Based on ANCOVA where age served as covariate.

^b^ coordinates are presented in the MNI atlas space in mm-s relative to the origin

^c^ Based on Talairach Daemon database atlases

*p<0.001

**Table 2:** Significant effects of biological gender on regional cortical structure^a^.

Cluster name	Cluster peak [x,y,z]^b^	Localization^c^	Number of voxels	Post hoc statistics F(1,30)	Post hoc statistics t(33)	Direction of effect
Gen#1	1.5, −40.5, 12	Right and Left Posterior Cingulate and Precuneus	239	25.18*	4.925^**^	Larger in males (#1–3)
Gen#2	30, −88.5, −13.5	Right Inferior Occipital Gyrus, Right Lingual Gyrus	33	15.17*	3.630^**^	
Gen#3	−15, −33, −10.5	Left Parahippocampal Gyrus, Left Cerebellum Anterior Lobe, Left Culmen	51	17.28*	4.680^**^	
Gen#4	37.5, 4.5, −15	Right Superior Temporal Gyrus	44	18.22*	4.840^**^	Larger in females (#4–6)
Gen#5	13.5, −15, −25.5	Right Brainstem, Pons	35	17.33^**^	4.756^**^	
Gen#6	−42, 10.5, −7.5	Left Superior Temporal Gyrus, Left Insula	31	20.43^**^	5.092^**^	

^a^ Based on ANCOVA where age served as covariate

^b^ coordinates are presented in the MNI atlas space in mm-s relative to the origin

^c^ Based on Talairach Daemon database atlases

*:p<0.05; **: p<0.001

**Table 3:** Regions of interest with significant GID × Biological gender interaction^a^.

Cluster name	Cluster peak [x,y,z]^b^	Localization^c^	Number of voxels	Post hoc statistics F(1,30)	CF v CM Post hoc t(16)	GF v GM Post hoc t(15)	CF v GF Post hoc t(16)	CM v GM Post hoc t(15)	CF v GM Post hoc t(19)	CM v GF Post hoc t(12)
GID×GEN#1	−34.5, −27,66	Left Precentral and Postcentral Gyri	304	22.39**	2.832	4.152**	2.400	4.664**	2.753	0.191
GID×GEN #2	−9. −57. 9	Left Posterior Cingulate. Left Precuneus. Left Calcarinus	32	15.24**	4.023**	2.075^NS^	1.960^NS^	3.528*	0.671^NS^	0.186^NS^
Larger local GM volume in subjects with female gender identity.										
GID×GEN #4	34.5. −72. −10.5	Right Occipital Lobe. Right Middle and Inferior Occipital Gyri. Right Fusiform Gyrus. Right Lingual Gyrus	123	19.86**	3.354*	3.177*	3.003	3.858*	0.041^NS^	0.443^NS^
GID×GEN #5	54. −75. −4.5	Right Inferior Temporal Gyrus	42	21.08**	2.455	3.885*	3.056*	3.264*	1.570^NS^	0.241^NS^

^a^ Based on ANCOVA where age served as covariate.

^b^ coordinates are presented in the MNI atlas space in mm-s relative to the origin

^c^ Based on Talairach Daemon database atlases.

*: p<0.0083, level of significance corrected according to Bonferroni.

**: p<0.001

^NS^ non significant (p≥0.05)

CF: biological female control; CM: biological male control; GF: biological female with GID; GM: biological male with GID

### Results of the post hoc analyses

Results of the post hoc analyses performed on the clusters for the GID effect indicated that local GM volume in healthy controls was higher in all significant regions compared with GID patients (229 voxels affecting the left cerebellum, 76 voxels affecting the right cerebellum, and a 32 voxel cluster including the left angular gyrus and the left inferior parietal lobule; Table 1).

In significant clusters of the Biological gender main effect, higher local GM volume was found in males compared to females in 3 of the clusters (239 voxels affecting the Right and Left Posterior Cingulate and Precuneus; 51 voxels affecting the Left Parahippocampal Gyrus, Left Cerebellum Anterior Lobe, Left Culmen; 33 voxels affecting the Right Inferior Occipital Gyrus, Right Lingual Gyrus), while the opposite direction of effect was found in the other clusters (44 voxels affecting the Right Superior Temporal Gyrus; 35 voxels affecting the Right Brainstem and Pons; 31 voxels affecting the Left Superior Temporal Gyrus, and Left Insula) (Table 2).

**Figure 3:**
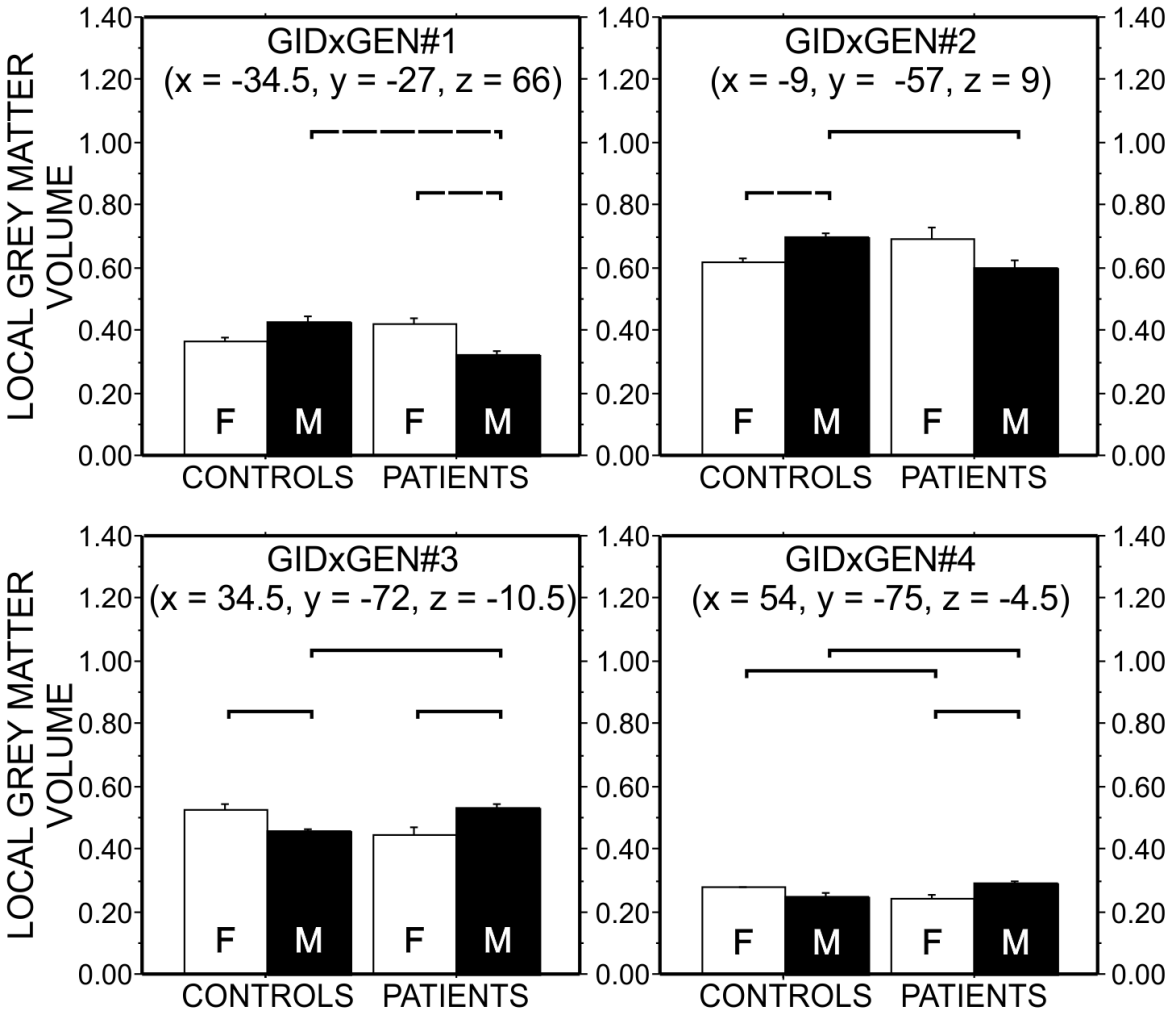
Post hoc analysis for GID × Biological gender contrast. Results of the post hoc analysis of the GID × Biological gender ANCOVA contrasts are shown. For this purpose voxel-wise GM intensity values (representing local GM volume), corresponding to the statistically significant clusters were extracted from the individual brains, then averaged within subject and submitted to a 2×2 ANOVA followed by all possible pair-wise comparisons of the control females, control males, FTM transsexuals and MTF transsexuals, using unpaired two-sample Student's tests between the groups. Each panel demonstrates the post hoc analysis results for an individual cluster (using the same labeling scheme as in Figure 2 previously). White bars show the local grey matter volume for biological females and black bars show the local grey matter volume for biological males. Dashed lines indicate significance level at p<0.001, while solid lines indicate significance level at p<0.0083 (Bonferroni corrected). GID: Gender Identity Disorder; FTM: female to male transsexual subject; MTF: male to female transsexual subject

The post hoc analyses of the significant clusters with GID × Biological gender interaction showed that transgender patients had significantly different regional GM volume from that of controls sharing their biological gender and did not differ significantly from that of controls sharing their gender identity in the examined clusters (Figures 3).

Specifically, in a 304 voxel sized cluster affecting the left pre- and postcentral gyri (including the somatosensory cortex and the primer motor cortex); a 32 voxel sized cluster affecting the left posterior cingulate, calcarine gyrus, and the precuneus, showed lower GM volume in MTF transgender patients and female controls compared to FTM transgender patients and male controls. The opposite direction of differences could be observed in a 123 voxel sized cluster in the right occipital lobe involving the middle and inferior occipital, the fusiform, and the lingual gyri, in a 42 voxel sized cluster affecting the right inferior temporal gyrus, where regional GM volume proved to be higher in MTF transgender patients and female controls compared with FTM transgender patients and male controls (please see Table 3 and Figure 3 for details).

## Discussion

Early studies investigating the brain structure of transgender patients were based on post mortem neuroanatomical methodology [3]–[5], but recently in vivo imaging studies gained importance. While the findings are diverse, the general trend of the results points towards a distributed pattern of neuroanatomical/structural differences subtending multiple brain areas of transgender subjects compared with controls from the same biological gender [6]–[14]. It has to be noted that direct comparison of the results of these studies is hampered by the different methodological approaches used.

We chose VBM as our method of investigation, and to our knowledge the present study is unique with respect to examining both FTM and MTF transsexuals with this approach. Hence, we were able to fully explore GID × Biological gender interactions, and we identified 4 clusters where local GM volume of transgender patients did not differ significantly from controls sharing their gender identity but were different from those sharing their biological gender. Specifically, in the area of the right middle and inferior occipital gyri, the fusiform, and the lingual gyri, the right inferior temporal gyrus, regional GM volume was higher in those subjects who had female gender identity (MTF transsexuals and female controls), while in the left pre-and postcentral gyri, the left posterior cingulate, calcarine gyrus and precuneus, regional GM volume was higher in those subjects with male gender identity (FTM transsexuals and male controls).

Previous studies that used VBM approach in a fashion somewhat similar to our study were limited to investigating the MTF type of GID. For example, Luders et al. [11] compared MTF transsexuals before cross-hormonal treatment (n = 24) to male (n = 30) and female controls (n = 30). Their VBM study showed that only the right putamen had significantly larger volume in MTF transsexuals compared with male control subjects, and was within the range found in females. Luders et al applied a relatively conservative approach when restricted their statistical maps to clusters ≥123 voxels, which, together with the fact that they examined MTF transgender subjects only, makes it difficult to directly compare our results. Nevertheless, there were only 2 brain areas that showed significant GID × Biological gender interaction and 1 brain area that showed significant GID effect at the cluster size limit of ≥123 voxels in our study: a cluster involving the left pre- and postcentral gyri (304 voxels) and a cluster (123 voxels) involving the right middle and inferior occipital gyri, the fusiform and lingual gyri and a cluster (229 voxels) involving the left cerebellum, respectively.

In another study also limited to MTF transsexuals Savic and Arver, [12] reported no “feminization” of any brain region with regard to structure. Nonetheless, certain brain areas (clusters ≥100 voxels) showed characteristic structural features in the transsexual group compared with both male and female control groups. Specifically, they found reduced thalamus and putamen volumes and increased GM volumes in the insular and inferior frontal cortex and in the right temporo-parietal junction (angular gyrus and superior temporal gyrus) in the transsexual group compared with both control groups. In our study, however only the angular gyrus (but in the left hemisphere) was affected among these areas, showing lower regional GM concentration in both FTM and MTF transgender subjects compared to controls, independent of their biological gender. When comparing the results reported by Savic and Arver to either our study or to other imaging studies in the literature of transsexualism, it has to be taken into consideration that their reported results were obtained from a solely nonhomosexual transsexual group of patients. The lack of real overlap between our and Savic and Arvers' findings, despite the very similar methodology used, might at least in part be explained by the difference of the sexual orientation of the two samples.

In a recent paper, the GM thickness of MTF transsexuals (n = 24) and age matched male controls (n = 24) was compared [13] and significant between group differences were reported in several areas including the right pre- and postcentral gyri, the left postcentral- and paracentral gyri, the right precuneus, the right inferior temporal, lingual and fusiform gyri [13]. In all of the reported regions, MTF transsexuals showed thicker GM compared to male controls and no areas were reported where controls showed thicker GM compared to MTF transsexuals [13]. Although they examined only biologically male subjects, while our study examined both male and female subjects, there is partial overlap between the affected brain areas found in our study and that of Luders et al [13]. Specifically, in both studies areas in the right inferior temporal, lingual, and fusiform gyri were affected and showed thicker/more concentrated GM in MTF transsexuals compared with male control subjects. Zubiaurre-Elorza et al's findings [14] are also consistent with these latter findings from both our and Luders et al's [13] study. These authors investigated both MTF (n = 18) and FTM transsexuals (n = 24) with MR volumetry, and compared the results with male (n = 29) and female control (n = 23) subjects [14]. They found that FTM transsexuals showed subcortical grey matter masculinization in the right putamen, while MTF transsexuals showed feminized cortical thickness data. Specifically, MTFs did not differ from female controls with regard to cortical thickness but differed from male controls in the regions of the lateral and medial orbito-frontal regions, the insula, and the medial occipital cortex of the right hemisphere [14]. Nonetheless, a caveat is needed when comparing cortical volumetry results with VBM because the latter approach can estimate either regional GM concentration or regional GM volume depending on the processing parameters; moreover due to normalization and modulation steps VBM represents both the volume and the shape of the local cortical sheet.

We found a robust difference between transsexuals and controls affecting both the anterior and posterior lobe of the left cerebellum (229 voxels), an effect that is independent from the biological gender. Such a difference in the cerebellar region has not been documented in the literature.

The regions found affected in our study are mainly involved in neural networks playing role in body perception, including memory retrieval, self-awareness, visual processing, body and face recognition and sensorimotor functions [27]–[30]. Findings from the studies of Savic and Arver [12] and Luders et al. [13] also implicated that these functional networks are affected. We must note, however, that it is still unclear how the observed structural differences might translate into functional differences. Furthermore, it is also unclear how the observed differences could be interpreted in terms of a causal chain: as parts of the neurobiological background or perhaps as the consequences of transsexualism [12].

### Methodological strengths and limitations

Our reported results have to be interpreted in light of certain limitations. Most importantly, given the limited availability of the patient population, and our strict exclusion criteria, the number of GID patients who finally underwent imaging was 17, which might be considered low, despite that this sample size is comparable to similar imaging studies in the literature [8]–[14]. The limited sample size may indeed limit statistical power, however it helps to pinpoint clear trends in the data that can be validated by further investigations.

We used VBM for investigating in vivo, regional structural brain differences between subjects with GID and controls. VBM is considered to be an objective and reliable measure for comparing local structural differences voxelwise, by pinpointing changes in the local composition of the cortex after discounting macroscopic differences in brain shape by means of normalization [19]. Nevertheless, VBM is hampered by certain methodological shortcomings that make it difficult to compare results across studies. First, the intensity data captured and analyzed with the VBM method cannot easily be translated into the “size” of a particular brain area, since it is rather a mixture of local volume and shape; moreover the results of segmentation heavily depend on data quality [31]. Second, the choice of processing parameters (e.g. whether to use standard or optimized VBM, whether to use modulation, whether to clean up GM boundaries, etc.), and the choice of statistical model can heavily influence results and interpretation, e.g. VBM on unmodulated GM compartments represents local grey matter concentration, while VBM on GM compartment modulated by the Jacobian normalization transformation represents the absolute volume [19], [31]. Interpreting VBM results may also be problematic, as it is often not easy to explain connection between grey matter volume and brain function.

Although there are no widely adopted standards in the VBM literature, we followed the guidelines recommended by Ridgway et al [25]. Due to the limited sample size, an exploratory whole brain analysis was performed first at p<0.001 uncorrected significance level, followed by post hoc-analyses at more stringent statistical criteria. We adopted this strategy since, on one hand, presenting uncorrected results does not necessarily limit the validity of the findings in case of VBM, as shown by Ashburner and Friston [19], who concluded that the validity of statistical tests based on uncorrected statistics was not severely compromised. On the other hand, in order to show that the significant clusters are indeed markedly different from the background, we presented unthresholded F-statistical maps, as well [25]. The post-hoc analyses were performed at the cluster level, hence being less susceptible to the nonstationarity of the smoothness of VBM data [19]. If a difference was found to be significant after the within-cluster averaging and the Bonferroni-corrected post-hoc comparisons, then it was supposed to be stronger than just a trend-effect.

The necessity and the proper way of cluster size thresholding is also an unsolved issue in the VBM literature. Based on the fact that the spatially variant smoothness distribution of VBM data limits the validity of such thresholding approaches [32], Ashburner and Friston stated that voxel-based extent statistic should not be used in VBM [19]. Nevertheless, most authors tend to utilize cluster size thresholding albeit with very different, and most often arbitrary parameters [33], [34]. The reason behind the popularity of such thresholding is that it can decrease the chance of Type I errors, even at lower than usual statistical thresholds, while it can also limit the graininess of the results. Based on our relatively low sample size, we decided to use cluster size thresholding. We set the threshold to 30 voxels, which is slightly more than 100 cubic millimeters. This choice is arbitrary, but it is in the range of previous papers [33], [34] and provides an easy to interpret size scale for the reader.

According to the pertinent literature, age is an important factor that might play a role in the inconsistent results of neuroimaging studies with regard to sex dimorphism [35], [36], i.e. age related changes might confound results. In order to minimize the age associated bias of our findings we included age matched controls in our sample which was originally from a young cohort with a narrow age range (17–38 years).

FTM and MTF transgender patients were both included in this study, which is rare in the literature. Including both MTF and FTM subjects in our analyses allowed us to differentiate between findings that are connected to the “patient” status (GID effect) from those findings that are connected to a GID × Biological gender interaction (i.e. that would suggest sex dimorphic changes). This statistical analytic approach is also unique in the pertinent literature.

## Conclusion

Our findings support the notion that structural differences exist between subjects with GID and controls from the same biological gender. We found that transsexual subjects did not differ significantly from controls sharing their gender identity but were different from those sharing their biological gender in their regional GM volume of several brain areas, including the left and right precentral gyri, the left postcentral gyrus (including the somatosensory cortex and the primary motor cortex), the left posterior cingulate, precueneus and calcarinus, the right cuneus, the right fusiform, lingual, middle and inferior occipital, and inferior temporal gyri. Additionaly, we also found areas in the cerebellum and in the left angular gyrus and left inferior parietal lobule that showed significant structural difference between transgender subjects and controls, independent from their biological gender.

There is only small number of studies in the field of structural imaging of transgender subjects and although the applied methods are different, sample sizes are modest and results are therefore yet inconclusive in details, significant structural differences were found between transgender patients and controls repeatedly and in some cases, in overlapping brain areas. These initial results, including the results of our study, need to be further replicated and refined in future studies on larger samples, as well as followed by functional imaging studies that might clarify how these structural differences impact the process of the disturbed evolution of gender identity and/or how disturbed gender identity affect brain structure and functions.
